# The Better By Moving study: A multifaceted intervention to improve physical activity in adults during hospital stay

**DOI:** 10.1177/02692155221105337

**Published:** 2022-06-14

**Authors:** Sven JG Geelen, Boukje M Giele, Cindy Veenhof, Frans Nollet, Raoul HH Engelbert, Marike van der Schaaf

**Affiliations:** 1 26066 Amsterdam UMC location University of Amsterdam, Rehabilitation Medicine, Amsterdam, Netherlands; 2 522567 Amsterdam Movement Sciences, Ageing & Vitality, Amsterdam, Netherlands; 3 Physical Therapy Research, Department of Rehabilitation, Physical Therapy Sciences & Sports, 8124 University Medical Centre Utrecht, Utrecht University, Utrecht, Netherlands; 4 Expertise Centre Healthy Urban Living, Research Group Innovation of Human Movement Care, 8119 University of Applied Sciences Utrecht, Utrecht, Netherlands; 5 Amsterdam Movement Sciences, Rehabilitation & Development, Amsterdam, Netherlands; 6 Centre of Expertise Urban Vitality, Faculty of Health, 10191 Amsterdam University of Applied Sciences, Amsterdam, Netherlands

**Keywords:** hospitalization, physical activity, adults, mobility, intervention

## Abstract

**Objective:**

‘Better By Moving’ is a multifaceted intervention developed and implemented in collaboration with patients and healthcare professionals to improve physical activity in hospitalized adults. This study aimed to understand if, how and why ‘Better By Moving’ resulted in higher levels of physical activity by evaluating both outcomes and implementation process.

**Design:**

Mixed-methods study informed by the Medical Research Council guidance.

**Setting:**

Tertiary hospital.

**Participants:**

Adult patients admitted to surgery, haematology, infectious diseases and cardiology wards, and healthcare professionals.

**Measures:**

Physical activity was evaluated before and after implementation using the Physical Activity Monitor AM400 on one random day during hospital stay between 8 am and 8 pm. Furthermore, the time spent lying on bed, length of stay and discharge destination was investigated. The implementation process was evaluated using an audit trail, surveys and interviews.

**Results:**

There was no significant difference observed in physical activity (median [IQR] 23 [12–51] vs 27 [17–55] minutes, *P* = 0.107) and secondary outcomes before-after implementation. The intervention components’ reach was moderate and adoption was low among patients and healthcare professionals. Patients indicated they perceived more encouragement from the environment and performed exercises more frequently, and healthcare professionals signalled increased awareness and confidence among colleagues. Support (priority, resources and involvement) was perceived a key contextual factor influencing the implementation and outcomes.

**Conclusion:**

Although implementing ‘Better By Moving’ did not result in significant improvements in outcomes at our centre, the process evaluation yielded important insights that may improve the effectiveness of implementing multifaceted interventions aiming to improve physical activity during hospital stay.

## Introduction

Hospitalized adult patients spend up to 87–100% of their time sitting or lying in bed.^1^ These low levels of physical activity during hospital stay have been observed in geriatric, surgical, medical and post-stroke patients.^1^ There is growing evidence that low levels of physical activity during hospital stay lead to adverse outcomes such as functional decline, prolonged length of hospital stay, institutionalization after discharge and mortality.^2–4^

Recent evidence revealed that interventions improving physical activity during a hospital stay can help prevent functional decline.^5–9^ However, patients are still put to bed when admitted,^10^ the hospital bed remains to be centrepiece,^11^ and very low physical activity levels continue to be observed in hospitalized patients all over the world.^1,12^ A discrepancy exists between what is known to prevent functional decline and what happens in current hospital care.

To bridge this gap, multifaceted interventions are needed that tackle multiple barriers and effect behavioural and cultural change with respect to physical activity during hospital stay.^13^ To date, several multifaceted interventions have been described and these show they can effectuate positive changes regarding the time patients spent lying in bed and sitting,^9,14^ mobility levels,^8^ functional decline,^7^ length of hospital stay^8,9^ and discharge home.^9,14^ Although these results are promising, most of them lack a process evaluation illuminating how these results occurred and how improvements can be made.

Process evaluations have been conducted concurrently or following a complex intervention to assess whether implementation was successful and to explore if, how and why the intervention had an impact.^15^ This insight could assist the interpretation of causality, offer suggestions for improving the implementation of such a multifaceted intervention and explain how the effect of this particular intervention on patient outcomes can be replicated in another context or setting. The United Kingdom Medical Research Council offers a framework consisting of three key functions: *implementation*, *mechanisms of impact* and *context* to guide a process evaluation.^15^ This framework has frequently been used to evaluate physical activity interventions.^16^

To improve physical activity in hospitalized adult patients admitted to the Amsterdam University Medical Centers, we developed and implemented a theory-driven, multifaceted intervention called ‘Better By Moving’ in close collaboration with patients and healthcare professionals.^17^ Insight in if, how and why the ‘Better By Moving’ intervention resulted in higher levels of physical activity will allow us to formulate recommendations for developing and implementing such a multifaceted intervention in another context or setting. Therefore, the aim of this study was to conduct a hybrid evaluation of ‘Better By Moving’, using an outcome and process evaluation to study both the intervention's effectiveness on physical activity and the implementation process in terms of *implementation*, *mechanisms of impact* and *context*. Furthermore, we assessed the effectiveness of time spent lying in bed, length of hospital stay and discharge destination.

## Methods

### Study design

This study used a mixed-methods evaluation study design, and the Medical Research Council guidance on process evaluations^15^ was used to guide the hybrid evaluation. To evaluate the effectiveness of the ‘Better By Moving’ intervention on patient outcomes, we collected quantitative data using before-after implementation cross-sectional measurements and longitudinal data from the hospital administrative system. To evaluate the implementation process, we concurrently collected quantitative and qualitative data using an audit trail (i.e. a strategy to trace the process), surveys and interviews. Study reporting followed the Standards for QUality Improvement Reporting Excellence (version 2.0).^18^ Ethical approval was granted by the Medical Ethics Committee of the Amsterdam UMC (W17_479 #18.003 and W19_213 #19.258), Amsterdam, The Netherlands. All patients and healthcare professionals provided written informed consent. The study protocol including details on design, implementation process and outcome measures has been published elsewhere.^17^

### Setting

This study was conducted within five hospital wards of the tertiary university hospital Amsterdam University Medical Centers, location Academic Medical Center, the Netherlands: two gastrointestinal and oncological surgery wards (January 2018 to January 2019), one hematology ward (August 2018 to August 2019), one infectious diseases ward (August 2018 to August 2019) and one cardiology ward (May 2019 to March 2020).

### The ‘Better By Moving’ multifaceted intervention

The primary goal of the ‘Better By Moving’ intervention was to improve physical activity in adult patients during hospital stay. We used a structured, step-by-step implementation plan according to the evidence-based Implementation of Change process model by Grol and Wensing.^17,19,20^

The first stage to develop the content of the ‘Better By Moving’ intervention was to assess the amount of time patients spent physically active and lying in bed in each ward and to identify the barriers and enablers to improve physical activity from the perspective of patients and healthcare professionals.^17^ The Theoretical Domains Framework to identify determinants of behaviour was used to support the exploration of barriers and enablers.^21^ The results have recently been published.^12,22^

Having established the barriers and enablers, the Behaviour Change Wheel^23^ was used to select intervention components (e.g. learning how to assist patients in physical activity) and implementation strategies (e.g. information brochures, clinical lessons). This was conducted by ward-specific working groups consisting of physician(s), physician assistant(s), nurses, nursing assistant(s), physical therapists, a program manager (SG) and a senior nurse or team leader.^21,23^ Convenience samples of patients, caregivers, team leaders and local policymakers provided occasional input. This process is outlined in Appendix 1 and resulted in eight intervention components targeting patients (Table 1) and 15 intervention components targeting healthcare professionals (Table 2). Most intervention components (*n* = 12) targeted the *Physical Opportunity* and *Social Opportunity* of patients and healthcare professionals. Seven intervention components targeted the *Physical Capability* and *Psychological Capability* and four intervention components targeted the patients’ and healthcare professionals’ *Reflective Motivation* (Tables 1 and 2).^23^

**Table 1.** Overview of intervention components targeting hospitalized patients.

COM-B domain	Intervention components *[including TDF domain that was addressed]*	Implementation strategies	Implemented at the following hospital wards:			
			A	B	C	D
Capability (physical)	No intervention components selected.					
Capability (psychological)	Increasing knowledge on the adverse health consequences as a result of physical inactivity during hospital stay. *[TDF domain Knowledge]*	➢ Provide newly admitted patients with information brochures informing them on the adverse outcomes related to physical inactivity	X	X	X	X
		➢ Place these information brochures on a visible place at the hospital ward	X	X	X	X
		➢ Create a website incorporating information on the adverse outcomes related to physical inactivity	X	X	X	X
		➢ Integrate the website in the hospital mailing for elective surgery patients	X	X		
	Increasing knowledge regarding what to do besides simply walking on the hallway. *[TDF domain Knowledge]*	➢ Provide newly admitted patients with information brochures informing them on what they could do in the hospital to remain physically active			X	X
		➢ Place these information brochures on a visible place at the hospital ward			X	X
Opportunity (physical)	Ensuring there is sufficient, adequate equipment. *[TDF domain Environmental Context and Resources]*	➢ Add a cycle ergometer with virtual reality screen to ensure alternatives are available to be physically active	X	X	X	
		➢ Add mobilizers (IV-pole which are more suitable to walk with) to ensure patients can ambulate independently	X	X		
	Organizing other activities to be physically active on the hospital ward. *[TDF domain Environmental Context and Resources]*	➢ Organize alternatives to be physically active: painting in the patient lounge				X
		➢ Organize alternatives to be physically active: shared lunch	X	X		
		➢ Organize alternatives to be physically active: exercise group			X	
	Making the harmful effects of physical inactivity visible. *[TDF domain Environmental Context and Resources]*	➢ Add prompts and cues to highlight the harmful effects of bedrest, such as posters, flyers and infographics	X	X	X	X
		➢ Reduce prompts and cues that promote inactivity	X	X	X	X
	Creating a stimulating hospital environment. *[TDF domain Environmental Context and Resources]*	➢ Add prompts and cues to reinforce physical activity behaviour, such as posters, infographics, whiteboards incorporating physical activity goals and mobility icons on the walls	X	X	X	X
Opportunity (social)	Creating a culture of increased physical activity. *[TDF domain Social Influences]*	➢ Provide patients with regular instructions on why the hospital advices patients not to wear pyjamas	X	X		
		➢ Incorporate the advice not to wear pyjama's during hospital stay in information brochures.	X	X		
		➢ Ensure activities are available for patients to participate in, such as: painting in the patient lounge, a breakfast buffet and a coffee break in the patient lounge.	X	X		X
Motivation (automatic)	No intervention components selected.					
Motivation (reflective)	Determining physical activity goals in collaboration with healthcare professionals. *[TDF domain goals]*	➢ Involve patients in determining physical activity goals on a daily basis	X	X	X	X
		➢ Prompt patients to participate in the determination of their physical activity goals	X	X	X	X
		➢ Provide feedback on the patients physical activity goals on a regular basis	X	X	X	X
		➢ Prompt patients to participate in the monitoring of their physical activity goals	X	X	X	X
		➢ Reflect with patients on their participation in the determination and monitoring of physical activity goals	X	X	X	X

COM-B: the Capability, Opportunity, Motivation – Behaviour system which forms the hub of the Behaviour Change Wheel; TDF: the Theoretical Domains Framework determinant framework; A: surgery ward #1; B: surgery ward #2; C: haematology ward; D: infectious diseases ward; E: cardiology ward.

**Table 2.** Overview of intervention components targeting healthcare professionals.

COM-B condition	Intervention components *[including TDF domain that was addressed]*	Implementation strategies	Implemented at the following hospital wards:			
			A	B	C	D
Capability (physical)	Learning how to use motivational interviewing. *[TDF domain Skills]*	➢ Organize clinical lessons to teach nurses and nursing assistants on motivational interviewing	X	X		X
		➢ Prompt nurses to practice motivational interviewing in patients who are unmotivated	X	X		X
		➢ Repeat the prompt for nurses to practice motivational interviewing in patients who are unmotivated	X	X		X
	Learning how to assist patients in physical activity. *[TDF domain Skills]*	➢ Organize clinical lessons to teach nurses and nursing assistants the skills to physically assist patients	X	X		X
		➢ Prompt nurses and nursing assistants during clinical care to practice the skills to physically assist patients	X	X		X
		➢ Repeat the prompt for nurses and nursing assistants during clinical care to practice the skills to physically assist patients	X	X		X
Capability (psychological)	Increasing knowledge on the adverse health consequences as a result of physical inactivity during hospital stay. *[TDF domain Knowledge]*	➢ Organize clinical lessons to inform healthcare professionals on the adverse outcomes related to physical inactivity	X	X	X	X
		➢ Organize clinical lessons to inform healthcare professionals on how improving physical activity relates to patient safety (i.e. fall avoidance)	X	X		X
		➢ Place infographics in the locker rooms informing all who pass on the adverse outcomes related to physical inactivity	X	X	X	
		➢ Add information on physical activity in the weekly hospital ward mailing lists				
	Providing the multidisciplinary team with a clear definition for ‘physical activity’. *[TDF domain Knowledge]*	➢ Organize hospital ward team meetings to discuss and agree on the definition of physical activity in their patient population	X	X		X
	Recognizing physical activity as a priority in routine clinical care. *[TDF domain Memory, Attention and Decision Processes]*	➢ Organize hospital ward team meetings to discuss, agree and reflect on physical activity as a priority in clinical care	X	X	X	X
		➢ Reflect with individual healthcare professionals on physical activity as a priority in clinical care	X	X	X	X
		➢ Add prompts and cues to remind healthcare professionals about encouraging patients in physical activity	X	X	X	X
Opportunity (physical)	Increasing transparency of the physical therapy recommendations. *[TDF domain Environmental Context and Resources]*	➢ Assess the factors why nurses and physicians might miss the physical therapy recommendations				X
		➢ Change the physical therapy reports according to these factors				X
		➢ Add prompts and cues to find the physical therapy reports				X
		➢ Demonstrate where physical therapy reports can be found				X
	Countering the lack of physical therapy consultations. *[TDF domain Environmental Context and Resources]*	➢ Integrate physical therapy consultations in protocols	X	X	X	
		➢ Integrate physical therapy consultations in routinely placed Electronic Medical Record orders	X	X	X	
	Making the harmful effects of physical inactivity visible. *[TDF domain Environmental Context and Resources]*	➢ Add prompts and cues to highlight the harmful effects of bedrest, such as posters, flyers and infographics	X	X	X	X
		➢ Reduce prompts and cues that promote inactivity	X	X	X	X
	Creating a stimulating hospital environment. *[TDF domain Environmental Context and Resources]*	➢ Add prompts and cues to reinforce physical activity behaviour, such as posters, infographics, whiteboards incorporating physical activity goals and mobility icons on the walls	X	X	X	X
	Ensuring there is sufficient, adequate equipment. *[TDF domain Environmental Context and Resources]*	➢ Add turners (bed-chair assistive support device) to improve bed-chair mobility	X	X		
		➢ Add mobilizers (IV-pole which are more suitable to walk with) to ensure patients can ambulate independently	X	X		
	Countering the perceived lack of time to encourage physical activity. *[TDF domain Environmental Context and Resources]*	➢ Analyze the factors influencing the lack of time	X	X	X	X
		➢ Assess how much time nurses lose on improving physical activity	X	X		
		➢ Find solutions to counter the factors influencing the lack of time	X	X	X	X
		➢ Provide feedback on the nurses’ behaviour, depending on the factors	X	X	X	X
		➢ Ensure the profit for improving physical activity is visible	X	X		
Opportunity (social)	Creating a culture of increased physical activity. *[TDF domain Social Influences]*	➢ Provide feedback on actual behaviour	X	X		X
		➢ Demonstrate the ideal behaviour in a situation of their choosing	X	X		
		➢ Discuss with the team of healthcare professionals common pitfalls with regards to encouraging physical activity	X	X		
		➢ Provide verbal rewards when the team of healthcare professionals collectively encourage physical activity as a team	X	X		
Motivation (automatic)	No intervention components selected.					
Motivation (reflective)	Responsibility for physical activity during hospital stay *[TDF domain Social/Professional Role and Identity]*	➢ Assess and review with physicians how they view their responsibility to improving physical activity	X	X	X	
		➢ Assess and review with physical therapists how they view their responsibility to improving physical activity	X	X	X	X
		➢ Assess and review with teams of healthcare professionals how they can share responsibility to improving physical activity	X	X	X	
		➢ Assess and review with teams of healthcare professionals how they view the role of the patient	X	X	X	X
	Creating confidence among healthcare professionals. *[TDF domain Beliefs about Capabilities]*	➢ Support nurses in encouraging physical activity	X	X	X	X
		➢ Demonstrate and practice how healthcare professionals can encourage patients	X	X	X	X
		➢ Invite physical therapists to encourage healthcare professionals	X	X	X	X
		➢ Provide occasional feedback on behaviour	X	X	X	X
		➢ Encourage healthcare professionals in their self-efficacy	X	X	X	X
	Determining physical activity goals in collaboration with patients. *[TDF domain Goals]*	➢ Assess the factors why nurses and physicians do not determine physical activity goals on daily basis	X	X	X	X
		➢ Prompt nurses and physicians to determine physical activity goals on a daily basis	X	X	X	X
		➢ Demonstrate how to use goal setting to improve physical activity	X	X	X	X
		➢ Provide tools to determine physical activity goals	X	X	X	X
		➢ Monitor the use of these tools	X	X	X	X
		➢ Provide feedback on the nurses’ and physicians use of these tools	X	X	X	X
		➢ Provide feedback on the nurses’ and physicians behaviour to use goal setting	X	X	X	X
		➢ Provide information on the consequences of inconsistently using goal setting (e.g. invalid measurements, failure to motivate patients)	X	X	X	X
		➢ Provide nurses and physicians with feedback on how to determine physical activity goals	X	X	X	X
		➢ Introduce environmental and social reminders to determine goals and use the tools	X	X	X	X
		➢ Incorporate goal setting in the electronic medical record	X	X	X	X

COM-B: the Capability, Opportunity, Motivation – Behaviour system which forms the hub of the Behaviour Change Wheel; TDF: the Theoretical Domains Framework determinant framework; A: surgery ward #1; B: surgery ward #2; C: hematology ward; D: infectious diseases ward; E: cardiology ward.

To implement the ‘Better By Moving’ intervention, each working group developed and executed an implementation plan for the intervention components they deemed feasible and effective at their hospital ward. The taxonomy of 93 Behaviour Change Techniques was used to specify implementation strategies.^23^ Throughout the implementation, working groups strived for optimal integration within routine hospital care. The implementation strategies linked to the intervention components are shown in Tables 1 and 2.

### Outcome evaluation

The primary outcome was the difference in amount of physical activity of patients before-after implementation. Physical activity was assessed on one random day during hospital stay between 8 am and 8 pm using the Physical Activity Monitor AM400.^24^ The Physical Activity Monitor AM400 is a 2-cm-wide coin attached to the ankle, and validly measures three-dimensional accelerations as a derivative for Metabolic Equivalent Tasks to determine the time patients spent physically active in minutes (>1.4 Metabolic Equivalent Tasks).^24,25^ Metabolic Equivalent Tasks express the energy cost of physical activities. Eligible patients are 18 years or older, can communicate in Dutch or English, and are admitted for at least 24 h. Exclusion criteria were: insufficient Dutch or English speaking and reading skills, inability to perform independent bed-to-chair transfer before hospital admission, delirium, obligatory bed rest, receiving end-of-life care and discharge before 12 am on the day of observations. Eligible patients were approached one or two days prior to observation in a random order using a computer-generated list based on the room numbers. Each patient could only be enrolled once throughout the study.

Secondary outcomes were time spent lying on bed, length of hospital stay and proportion of patients discharged home instead of a temporarily institution. Time spend lying in bed was assessed over the same time period as the primary outcome using a behavioural mapping protocol.^26^ Length of stay and proportion of patients discharged home were assessed in all patients admitted for three days or longer to one of the hospital wards using an Interrupted Time Series approach over 30 months (i.e. 12 months before, 12 months during and 6 months after implementation).^27^ This approach allowed us to assess the effect over time by comparing the slopes (i.e. trends) before-during-after implementation and to assess the immediate effect by comparing the level changes at implementation start (month 0) and stop (month 13).^27^ Discharge destination was categorized as (1) home with or without homecare, and (2) discharged to a temporary institution (i.e. temporary nursing home, geriatric rehabilitation center, or medical rehabilitation center). Discharges to permanent nursing homes, other hospitals, end-of-life care facilities or patients who passed away were omitted. The data were obtained as de-identified data from the hospital administrative system.

### Process evaluation

**Figure 1.**
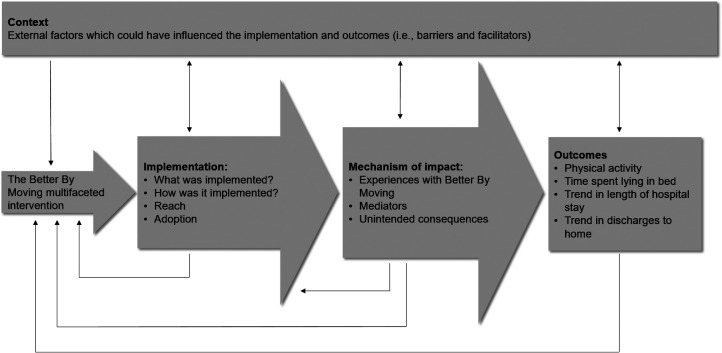
Key functions of the ‘Better By Moving’ intervention process evaluation and relations among them (after UK Medical Research Council guidance.^15^)

Process outcomes were defined as *implementation*, *mechanisms of impact* and *context*^15,17^ (Figure 1):

- *Implementation:* (1) an audit trail detailing which and how intervention components were implemented and (2) a questionnaire for patients and healthcare professionals containing closed- and open-ended items before and after implementation assessing reach (i.e. the extent to which patients and healthcare professionals come into contact with the intervention components and adoption (i.e. uptake of the intervention components).
- *Mechanism of impact:* (1) the questionnaire for patients contained five-point Likert scale items to assess the perceived encouragement from healthcare professionals and hospital environment, the perceived need for information, the frequency patients indicate they perform exercises and the perceived self-efficacy to perform mobility activities before and after implementation, and (2) the questionnaire for healthcare professionals contained closed- and open-ended items exploring their perspective on the mechanisms after implementation.
- *Context:* (1) an audit trail keeping track of all contextual factors that may have influenced the implementation process, and (2) open-ended survey questions exploring their perspective on the contextual influences after implementation.

For the process evaluation, all hospitalized patients were asked to complete the questionnaire one day after the day of observation. Furthermore, healthcare professionals employed for at least 70% of full-time equivalent at one of the five wards were asked to complete the questionnaire after implementation. After the questionnaire, we conducted semistructured interviews with healthcare professionals to provide a more in-depth understanding of the survey responses concerning all three process outcomes. We aimed to include a heterogeneous group with respect to working group participation, working experience and profession.

### Data analysis

No a priori sample size calculations were performed. Financial and logistic resources allowed us to include a pragmatic sample with an estimated size of *n* = 110 before implementation and *n* = 110 after implementation. Given this sample size, we were able to detect an effect size of 0.38 or higher for the primary outcome variable (two groups t-test of equal means; α = 0.05; 1–β = 0.80; nQuery 8, Statistical Solutions Ltd).^17^

Descriptive statistics were derived to describe the patient characteristics. Normality was evaluated with the Kolmogorov–Smirnov test and Q–Q plots. A negative binomial regression model was used to assess the difference in physical activity before-after implementation, with the presence of urinary catheter used as a covariate.^12,17^ An analysis of covariance was performed to assess the difference in time spent lying on bed and included the same covariate. In the published protocol,^17^ independence in mobility was also mentioned as covariate; however, due to a significant difference (*P* = 0.026) in independence of mobility between the two groups, we could not assume independence of the covariate and effect. An interrupted time series analysis was used to evaluate the changes in length of hospital stay and discharge destination using a monthly interval. All quantitative analyses were conducted using IBM SPSS Statistics version 26 (IBM Corp). Parameter estimates were expressed using a 95% confidence interval (95%CI), and results were considered significant if *P* < 0.05.

Qualitative data were analyzed using the thematic analysis approach as described by Braun and Clark.^17,28^ The research team participated in refining the preliminary themes and member checking was used with the participants to ensure the credibility of the data analysis. MAXQDA Analytics Plus 2018 (VERBI Software) supported the analysis.

## Results

Due to the COVID-19 crisis, the implementation of the ‘Better By Moving’ intervention in the Cardiology ward was put on hold in March 2020 and, as a result, the cardiology ward is not included in the hybrid evaluation.

### Outcome evaluation

#### Participant characteristics

In total, 88 and 85 patients, respectively, were included before-after implementation. Patient characteristics of both groups are presented in Table 3. No significant differences in patient characteristics before-after implementation were observed (*P* > 0.05), except for independency in basic mobility activities (*n* = 54 [61.4%] before vs *n* = 66 [77.6%] after, *P* = 0.026). For the evaluation of length of stay and discharge destination, the de-identified dataset comprised of 2584 patients before, 2454 patients during and 1229 patients after implementation. No time-varying confounders were identified (e.g. significant changes in admission and discharge procedures).^27^

#### Primary and secondary outcomes

After implementation, there was no significant difference observed in physical activity between 8 am and 8 pm compared to before implementation (median [IQR] 23 [12–51] minutes before vs 27 [17–55] minutes after, *P* = 0.107) (Appendix 2). No significant difference was observed in the time spent lying in bed between 8 am and 8 pm (72.6% before vs 67.4% after, *P* = 0.115) (Appendix 2). The trend for length of stay did not change after starting and completing the implementation. Additionally, there were no significant level changes at the start and after completing the implementation (Appendix 3). The trend for the proportion of patients discharged home did not change after starting and completing the implementation (*P* > 0.05). Additionally, there was also no significant level change at the start and after completing the implementation (Appendix 3).

**Table 3.** Before-after implementation differences in patient characteristics.

	Before	After	
Characteristic	*n* = 88	*n* = 85	*P* value
Female, *n* (%)	42 (47.7%)	41 (48.2%)	0.115
Age (years), median (IQR)	60 (46–69)	63 (50–72)	0.154
Charlson Comorbidity Index, median (IQR)	3 (1–6)	3 (1–4)	0.118
Number of days between day of admission and day of observation, median (IQR)	8 (3–11)	6 (3–12)	0.775
Length of stay (days), median (IQR)	13 (7–25)	12 (8–22)	0.890
Independent in basic mobility^a^, *n* (%)	54 (61.4%)	66 (77.6%)	0.026
Urine catheter^b^, *n* (%)	26 (29.5%)	24 (28.2%)	0.850

IQR: interquartile range.

^a^ Score of 20 when using questions 1–5 Activity Measure for Post-Acute Care (AM-PAC) Basic Mobility.

^b^ Urine catheter presence.

### Process evaluation

#### Participant characteristics

In total, *n* = 87 of the *n* = 88 patients completed the survey before and *n* = 81 of the *n* = 85 after implementation. Sixty-seven of 144 eligible (47%) healthcare professionals working at the moment of survey distribution completed the survey (*n* = 16 at surgery #1, *n* = 13 at surgery #2, *n* = 21 at haematology, *n* = 17 at infectious diseases). They were working as a physician (*n* *=* 4), nurse or nurse assistant (*n* *=* 61) or physical therapist (*n* *=* 2). The median (IQR) working experience was three years (2–12). Three team leaders, three senior nurses, two physicians, one nursing assistant and three nurses participated in semistructured interviews.

#### Implementation

Tables 1 and 2 show which intervention components were implemented and how they were implemented per hospital ward. In summary, clinical lessons to increase knowledge (attendance 61% [*n* *=* 36, missing *n* *=* 8]) and to improve skills (hands-on training; attendance 23% [*n* *=* 9, missing *n* *=* 28]) were provided. Furthermore, a cycle ergometer with virtual reality, a turntable, and ambulation-friendly IV poles were purchased. Posters, flyers and infographics were placed and, where possible, care pathways incorporated an increased mobilization regime, structured physical therapy consultations and daily physical activity goals. A person-centered communication board focusing on mobility was implemented, and a mobility scale was implemented into the electronic medical record to monitor these goals. Bedside teaching and individual coaching were conducted by physical therapists to improve skills and increase confidence.

Reach among patients varied from 26% (*n* = 21) to 78% (*n* = 62, missing *n* *=* 1) with the lowest reach for the component encouraging patients to set physical activity goals with healthcare professionals and the highest for the component aiming to create a stimulating hospital environment (e.g. posters, infographics and mobility icons on the walls). According to healthcare professionals reach was sufficient, except for the low-reach components: ‘learning how to use motivational interviewing techniques’, ‘learning how to assist patients in physical activity’, ‘recognizing physical activity as a priority in clinical care’ and ‘counteracting the perceived lack of time to encourage physical activity’.

Adoption among patients who were reached varied from 19% (*n* = 4) to 57% (*n* *=* 33) with the lowest reach for the component encouraging patients to set physical activity goals with healthcare professionals and the highest for the component aiming to organize activities on the hospital ward for patients. According to healthcare professionals adoption was low to moderate, depending on the type of component. Adoption was highest for components targeting physical therapists, equipment and ward environment. By contrast, components requiring daily attention or aiming to change every day routines were scarcely adopted.

#### Mechanisms of impact

In total, 43.8% (*n* = 35, missing *n* *=* 1) of the patients were aware of the ‘Better By Moving’ intervention, and 74.1% (*n* *=* 60) was satisfied with how physical activity was encouraged after implementation. Furthermore, patients perceived significantly more encouragement from the hospital environment (mean 2.93 [SD = 1.054] before vs 3.36 [0.971] after on a five-point Likert scale, *P* = 0.007) and indicated they exercised more frequently (mean 3.17 [SD = 1.287] before vs 3.59 [1.249] after on a five-point Likert scale, *P* = 0.032) after implementation. No significant before-after differences were observed regarding all other survey items.

The majority (59.7%, *n* = 40) of the healthcare professionals believed that the intervention components of the ‘Better By Moving’ intervention combined resulted in improved levels of physical activity in patients at their ward. These healthcare professionals indicated that the ‘Better By Moving’ intervention increased the healthcare professionals’ awareness regarding the benefits of physical activity, leading to more frequent encouragement of patients to be physically active. Additionally, confidence in encouraging physical activity was improved. A minority (40.3%, *n* = 27) of healthcare professionals believed that the ‘Better By Moving’ intervention needed more time to influence patient's physical activity behaviour as currently, they still encounter difficulties in motivating patients to be physically active.

#### Context

We identified five barriers influencing the implementation and outcomes from the perspective of healthcare professionals. Firstly, the inability of nurses to consistently participate in the working groups. Secondly, the lack of active involvement of physicians in the working group. Thirdly, the lack of resources to facilitate working group participants to spend more time on implementing intervention components or to finance more substantive changes to the wards’ environment. Fourthly, an imminent renovation or relocation of the wards, preventing more permanent environmental changes from being allowed. Lastly, the working group participants at some wards perceived a lack of support from leadership and the multidisciplinary team, caused by the multiplicity of different projects.

A facilitator was the availability of a project manager to manage, facilitate and keep an overview of the quality improvement process during all phases at the different wards. The multidisciplinary approach was also commonly noted as a facilitator to develop intervention components that were not only related to one profession. Furthermore, the extensive exploration of barriers and enablers and the bottom-up approach to select and implement intervention components meeting the needs of the wards were noted as facilitators. Both contributed to making the healthcare professionals feel heard and to creating ownership among working group participants. They also indicated the project did not increase the nursing workload. Support of the leadership and involvement of patients and caregivers in selecting intervention components were specifically reported as facilitators by workgroup participants. Lastly, having small multidisciplinary projects focusing on improving physical activity prior to the ‘Better By Moving’ intervention and aligning intervention components with routine clinical care or other running projects (e.g. the involvement of patients’ caregivers in postoperative care) were perceived as facilitators.

## Discussion

The aim of this study was to conduct a hybrid evaluation of ‘Better By Moving’, a newly developed theory-informed multifaceted intervention to improve physical activity in hospitalized adult patients. The findings of our outcome evaluation indicate that implementing the ‘Better By Moving’ intervention on four different wards of the Amsterdam University Medical Centers was not associated with significant improvement in physical activity, reduction of time spent lying in bed or length of hospital stay or increase in discharges home. These results are in contrast with previous studies that reported improved patient outcomes after implementing a multifaceted intervention during hospital stay.^7–9,14,29^ To obtain insight in why the ‘Better By Moving’ intervention was not associated with significant improvement in physical activity and secondary outcomes, we concurrently conducted a process evaluation. Based on this process evaluation, we hypothesize that moderate reach and low adoption contributed to the lack of effectiveness of the ‘Better By Moving’ intervention. By using a bottom-up co-creational approach to select, tailor and implement the intervention components,^17^ we intended to establish ownership among healthcare professionals and leadership.^30^ Apparently, this was not achieved, likely due to the lack of support in terms of priority, resources and involvement, both at staff- and ward level. Other reasons for different outcomes compared to previous studies may be the amount and variety in intervention components,^7–9,14,29^ difference in outcome evaluation (e.g. procedures, analyses),^7–9,14,29^ difference in patient population^7,8,29^ or difference in context (e.g. local hospital admissions and discharge policies).^7,8,29^

To date, two previous multifaceted interventions to improve in-hospital physical activity included a process evaluation^29,31^ and only one of these evaluated reach and adoption of their intervention components.^31^ With reach and adoption varying from 54% to 86%,^31^ they observed a significant decrease in time spent lying in bed and number of discharges to a rehabilitation setting after implementation.^14^ This contrasts with our findings indicating that significant improvements in patient outcomes are likely depending on the level of reach and adoption of each intervention component.^15^ Therefore, we recommend that reach and adoption should be monitored during implementation. A recent study performed by Khadjesari et al.^32^ provides guidance on how to measure reach and adoption validly and reliably during implementation.

Further, statistically significant differences in several *mechanisms of impact* were observed: more patient encouragement from the hospital environment, patients indicating they exercised more frequently, and increased awareness and confidence among healthcare professionals. A new implementation cycle would be needed to determine how to sustain these changes and assess why these mechanisms did not result in more physical activity and better patient outcomes.^20^ Two previous studies in which similar multifaceted interventions were implemented also illustrated that going through an implementation cycle once does not necessarily result in positive outcomes in every hospital ward.^9,14^ This suggests that multifaceted interventions can benefit from a more flexible and iterative approach in general wherein healthcare professionals are encouraged to continually evaluate, and, where necessary, adapt the intervention and implementation in short cycles.

Even though it is commonly assumed that multifaceted interventions lead to more effective changes when compared to single-component interventions, compelling evidence is still lacking.^33^ Given the moderate reach and low adoption, we agree with the suggestion made by previous researchers that fewer components with a clear hierarchical structure may help to increase effectiveness^31,33^ because a narrow focus ensures that more attention and effort can go to implementing the intervention components (i.e. improving reach and adoption). An example of such intervention is the multifaceted intervention study conducted by Cohen et al.^7^ focusing on implementing the 900-step mobility goal to prevent hospitalization-associated functional decline among older adults. Video clips, in-personal communication, brochures, posters and staff-training were all used to support achieving this walking-dose benchmark in as many patients as possible.

Finally, implementing a multifaceted intervention may have different effects in different contexts even if its implementation does not vary.^15^ Through the implementation of the ‘Better By Moving’ intervention in the Amsterdam University Medical Centers, we identified five contextual barriers and eleven contextual facilitators, with the level of support in terms of priority, resources and involvement—both at staff- and ward level—appearing to be the common denominator. Context includes anything external to the intervention,^15^ but this does not necessarily mean that contextual factors cannot be influenced. This has recently been highlighted by Geerligs et al.,^30^ who recommend to consider factors relating to the staff and system (e.g. ward) as active components that can be influenced during intervention development and implementation.

### Strengths and limitations of the study

A major strength of this study is the integration of outcome and process evaluation.^15^ Another strength of this study was the combination of different theoretical approaches used to address various implementation and evaluation challenges in the ‘Better By Moving’ intervention.^15,19,21,23^ Lastly, an important strength of this study is the implementation of usual care in a heterogeneous hospital population.

This study also has some limitations. Firstly, our uncontrolled before-after study design does not allow us to conclude direct causation.^34^ Secondly, the limited power of our before-after evaluation of physical activity and time spent lying in bed may have caused a type II error (i.e. false negative results).^17^ Thirdly, although the ‘Better By Moving’ intervention may have been effective on individual hospital wards, lack of statistical power in this quality improvement project did not allow a ward-specific analysis. Fourthly, this is a single-site study in a university hospital in the Netherlands, limiting the generalizability of the results.

## Conclusions

Although implementing the ‘Better By Moving’ intervention did not result in significant improvements in physical activity and secondary outcomes at our center, the process evaluation of ‘Better By Moving’ yielded valuable information that may improve the effectiveness of implementing future multifaceted interventions aiming to improve physical activity during hospital stay. Future research should focus on investigating the effectiveness of using a more flexible and iterative approach to improve physical activity during hospital stay.

**Clinical messages**

- Multifaceted interventions to improve in-hospital physical activity are more likely to be successful when they include a limited amount of components with a clear hierarchical structure, implementation teams closely monitor reach and adoption during implementation, and implementation teams are sufficiently supported in terms of priority, resources and involvement.

## Appendix

**Appendix 1.** An overview of the perceived barriers and enablers with linked intervention functions and BCTs.

COM-B domain	TDF domain	Barriers and enablers	Intervention function	Policy categories	Behaviour change techniques
Capability (physical)	Skills	**Patients** lack the skills to ambulate with functional restraints	Training	Service provision	4.1 Instruction on how to perform the behaviour 8.1 Behaviour practice/rehearsal 8.3 Habit formation
	Skills	**Nurses and nursing assistants** lack the skills to physically assist patients	Training	Service provision	4.1 Instruction on how to perform the behaviour 8.1 Behaviour practice/rehearsal 8.3 Habit formation
Capability (psychological)	Knowledge	**Healthcare professionals** differ in how they define physical activity	Education	Communication/marketing; Guidelines	5.1 Information about health consequences 9.1 Credible source
	Knowledge	**Nurses and physicians** lack knowledge on how to formulate physical activity goals	Education	Communication/marketing; Service provision	2.2 Feedback on behaviour 8.1 Behaviour practice/rehearsal 8.2 Behaviour substitution 8.4 Habit reversal
	Knowledge	**Patients** are not aware of ‘what to do’ and ‘where to go’	Education	Communication/marketing	7.1 Prompts/cues
	Knowledge	**Patients** are not aware of the physical inactivity-related complications	Education	Communication/marketing	5.1 Information about health consequences
	Knowledge	**Nurses** lack knowledge on how to motivate unmotivated patients	Education	Communication/marketing; Service provision	2.2 Feedback on behaviour 8.1 Behaviour practice/rehearsal 8.2 Behaviour substitution 8.4 Habit reversal
	Knowledge	**Nurses and nursing assistants** lack knowledge about safety and physical activity	Education	Communication/marketing; Service provision	2.2 Feedback on behaviour 5.3 Information about social and environmental consequences
	Memory, Attention and Decision processes	Encouraging physical activity is not a high priority to **nurses and physicians**	Enablement; Training	Guidelines; Regulation	1.7 Review the outcome goal 2.2 Feedback on behaviour 3.1 Social support (unspecified) 5.1 Information about the health consequences 8.3 Habit formation
	Memory, Attention and Decision processes	**Patients** are rarely reminded to be physically active	Environmental restructuring	Environmental/social planning	7.1 Prompts/cues 12.5 Adding objects to the environment
	Behavioural Regulation	**Patients** do not participate in the self-monitoring of in-hospital physical activity	Enablement; Modelling	Environmental/social planning	1.4 Action planning 2.2 Feedback on behaviour 2.3 Self-monitoring of behaviour 7.1 Prompts/cues 12.5 Adding objects to the environment
	Behavioural Regulation	**Nurses and physicians** do not determine physical activity goals on a structural basis	Enablement; Modelling	Environmental/social planning; Regulation	1.2 Problem-solving 1.4 Action planning 2.1 Monitoring of behaviour by others without feedback 2.2 Feedback on behaviour 7.1 Prompts/cues 12.5 Adding objects to the environment
	Behavioural Regulation	Physical therapy recommendations are often not followed	Education; Modelling	Communication/marketing	1.2 Problem-solving 2.2 Feedback on behaviour
Opportunity (physical)	Environmental Context and Resources	Sufficient, adequate equipment that supports **patients** in physical activity is rare	Environmental restructuring	Environmental planning	12.5 Adding objects to the environment
	Environmental Context and Resources	Sufficient, adequate equipment that supports **healthcare professionals** in encouraging physical activity is rare	Environmental restructuring	Environmental planning	12.5 Adding objects to the environment
	Environmental Context and Resources	**Nurses** lack time to invest in encouraging physical activity	Training; Enablement	Guidelines; Service provision	1.2 Problem-solving 2.2 Feedback on behaviour 3.1 Social support 8.1 Behaviour practice/rehearsal 15.2 Mental rehearsal of successful performance
	Environmental Context and Resources	There is a lack of physical therapy consultations	Environmental restructuring	Guidelines; Regulation	7.8 Associative learning 12.1 Restructuring the physical environment
	Environmental Context and Resources	The hospital environment entices physical inactivity	Environmental restructuring	Environmental planning	7.1 Prompts/cues 7.3 Reduce prompts/cues 12.1 Restructuring the physical environment 12.5 Adding objects to the environment
Opportunity (social)	Social influences	**Patients** are not regularly encouraged by nurses	Enablement; Modelling	Environmental/social planning	1.2 Problem-solving 3.1 Social support 6.1 Demonstration of the behaviour 10.5 Social incentive 10.9 Social reward
	Social influences	**Patients** are rarely encouraged by physicians	Enablement; Modelling	Environmental/social planning	1.2 Problem-solving 3.1 Social support 6.1 Demonstration of the behaviour 10.5 Social incentive 10.9 Social reward
	Social influences	**Healthcare professionals** rarely involve family to encourage patients	Enablement	Communication/marketing; Guidelines	1.4 Action planning 6.1 Demonstration of behaviour 6.3 Information about others’ approval 7.1 Prompts/cues 12.5 Adding objects to the environment
	Social influences	**Healthcare professionals** rarely assess and discuss physical activity during interprofessional meetings	Enablement; Education	Regulation; Environmental/social planning	1.9 Commitment 2.2 Feedback on behaviour 7.1 Prompts/cues 12.5 Adding objects to the environment
Motivation (automatic)	Reinforcement	There is no reinforcement for walking	Incentivization; Environmental restructuring	Environmental/Social planning	12.5 Adding objects to the environment 14.5 Rewarding completion
	Reinforcement	Using the cycle ergometer on the hospital ward is boring	Incentivization; Environmental restructuring	Environmental/Social planning	12.5 Adding objects to the environment 14.5 Rewarding completion
	Reinforcement	The harmful effects of bedrest are invisible to **patients**	Incentivization; Environmental restructuring	Environmental/Social planning; Communication/marketing	12.5 Adding objects to the environment
	Reinforcement	The harmful effects of bedrest are rarely visible to **nurses and physicians**	Incentivization; Environmental restructuring	Environmental/Social planning; Communication/marketing	12.5 Adding objects to the environment
	Emotion	None identified			
Motivation (reflective)	Social/Professional Role and Identity	**Patients** rarely take responsibility for physical activity	Education; Persuasion	Communication/marketing;	2.2 Feedback on behaviour 4.1 Instruction on how to perform the behaviour
	Social/Professional Role and Identity	**Patients** wear pyjama's during hospital stay	Education; Persuasion	Communication/marketing;	2.2 Feedback on behaviour 4.1 Instruction on how to perform the behaviour 12.5 Adding objects to the environment
	Social/Professional Role and Identity	**Physicians** do not feel sufficiently responsible for encouraging physical activity	Education; Persuasion	Guidelines	1.2 Problem-solving 1.5 Review behaviour goal
	Social/Professional Role and Identity	**Physical therapists** do not feel sufficiently responsible for encouraging physical activity	Education; Modelling	Regulation; Guidelines	1.2 Problem-solving 1.5 Review behaviour goal
	Social/Professional Role and Identity	**Healthcare professionals** have no shared responsibility for improving physical activity	Modelling	Communication/marketing;	1.2 Problem-solving 1.5 Review behaviour goal
	Beliefs about capabilities	**Patients** have no confidence in general to be physically active	Persuasion; Enablement	Environmental/Social planning	12.6 Body changes 15.1 Verbal persuasion of capacity 15.2 Mental rehearsal of successful performance
	Beliefs about capabilities	**Patients** feel impaired by physical complaints	Enablement	Environmental/Social planning	12.6 Body changes 15.1 Verbal persuasion of capacity
	Beliefs about capabilities	**Healthcare professionals** have rarely confidence that they are able to encourage physical activity	Education; Modelling	Communication/marketing; Service provision	6.1 Demonstration of the behaviour 8.1 Behaviour practice/rehearsal 9.1 Credible source 15.1 Verbal persuasion of capacity
	Optimism	None identified			
	Intentions	**Patients** are not motivated to be physically active in general	Education; Persuasion;	Communication/marketing; Environmental/Social planning	1.1 Goal setting 1.2 Problem-solving 1.4 Action planning 7.1 Prompts/cues
	Intentions	**Nurses** are not aware that improving physical activity might save time on the long term	Education; Persuasion	Communication/marketing	2.2 Feedback on behaviour 2.6 Biofeedback 10.1 Material incentive (behaviour) 15.3 Focus on past success
	Goals	**Healthcare professionals** rarely use goal setting to encourage patients	Education; Modelling; Enablement	Service provision; Communication/marketing; Guidelines	2.2 Feedback on behaviour 5.3 Information about social and environmental consequences 6.1 Demonstration of the behaviour 7.1 Prompts/cues 12.5 Adding objects to the environment
	Beliefs about Consequences	**Patients** are afraid to ambulate because they relate it to falling	Education	Communication/marketing	2.2 Feedback on behaviour 8.1 Behaviour practice/rehearsal 15.1 Verbal persuasion about capacity

COM-B: the Capability, Opportunity, Motivation – Behaviour system which forms the hub of the Behaviour Change Wheel; TDF: the Theoretical Domains Framework determinant framework.

## Appendix

**Appendix 2.** Before-after implementation differences in physical activity and time spent lying in bed between 8 am and 8 pm.

Outcome	Before	After	*P* value
	*n* = 88	*n* = 85	
Physical activity in minutes, median (IQR)	23 (12–51)	27 (17–55)	0.107
Percentage of time spent lying in bed, mean (SD)	72.6% (19.3)	67.4% (23.4)	0.115

IQR: interquartile range; SD: standard deviation.

## Appendix

**Appendix 3.** Trend and level changes over time in length of hospital stay and proportion of patients discharged home.

Outcome	β	Standard error	*P* value
**Length of hospital stay**			
Change in trend after starting the implementation	<0.001	0.006	0.998
Change in level at the start the implementation	−0.020	0.042	0.63
Change in trend after completing the implementation	0.005	0.013	0.73
Change in level after completing the implementation	0.056	0.056	0.72
**Discharges home**			
Change in trend after starting the implementation	0.013	0.035	0.72
Change in level at the start the implementation	−0.008	0.244	0.97
Change in trend after completing the implementation	0.029	0.075	0.70
Change in level after completing the implementation	−0.089	0.319	0.78
